# Environmental stress influences Malesian Lamiaceae distributions

**DOI:** 10.1002/ece3.9467

**Published:** 2022-11-02

**Authors:** Liam A. Trethowan, Camilla Arvidsson, Gemma L. C. Bramley

**Affiliations:** ^1^ Herbarium Kew Royal Botanic Gardens Kew London UK; ^2^ Department of Biosciences University of Exeter Exeter UK

**Keywords:** biogeography, dispersal, macroecology, Malesia, mints, stress tolerance, Wallacea

## Abstract

Dual effects of spatial distance and environment shape archipelagic floras. In Malesia, there are multiple environmental stressors associated with increasing uplands, drought, and metal‐rich ultramafic soils. Here, we examine the contrasting impacts of multifactorial environmental stress and spatial distance upon Lamiaceae species distributions. We used a phylogenetic generalized mixed effects model of species occurrence across Malesia's taxonomic database working group areas from Peninsular Malaysia to New Guinea. Predictor variables were environmental stress, spatial distance between areas and two trait principal component axes responsible for increasing fruit and leaf size and a negative correlation between flower size and plant height. We found that Lamiaceae species with smaller fruits and leaves are more likely to tolerate environmental stress and become widely distributed across megadiverse Malesian islands. How global species distribution and diversification are shaped by multifactorial environmental stress requires further examination.

## Graphical abstract

Environmental stress varies across the megadiverse tropics. Here, we show how stress from soil, altitude and drought jointly shape mint species distributions across the islands of Malesia.

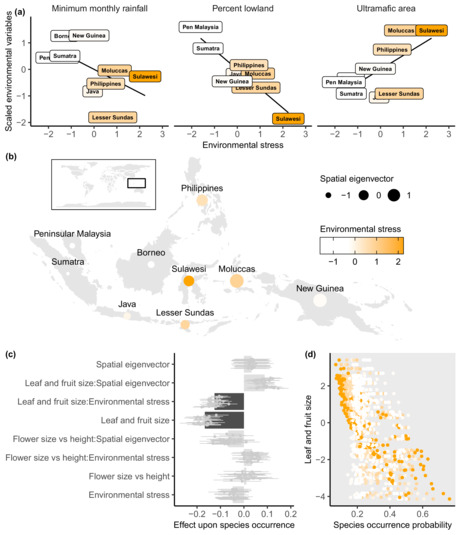

## 1 INTRODUCTION

The relative influence of either dispersal distances or the environment upon the occurrence of species in communities across large scales is unclear (Bemmels et al., 2018; Carvajal‐Endara et al., 2017; Dexter et al., 2017; Ibanez et al., 2018). The geographic or spatial distance between sites consistently shapes the composition of communities (Wallace, 1869). In general, the shorter the dispersal distance between communities the more species they share (Condit et al., 2002; Nekola and White, 1999). Similar environments are also more likely to share species (Weigelt et al., 2015; Whittaker, 1960). However, in archipelagos, examples of species distributions shaped more by the environment than spatial distance between islands are less frequent (König et al., 2021; Carvajal‐Endara et al., 2017; Giarla et al., 2018; Ng et al., 2017). A global analysis of oceanic islands found that altitude can significantly shape which plants are present (König et al., 2021). In the Galapagos, climate was found to be the main driver of island floras (Carvajal‐Endara et al., 2017). Malesia, the phytogeographical region stretching from Peninsular Malaysia to New Guinea, offers another opportunity to compare the effects of dispersal distances and the environment upon archipelagic species distributions. The region houses more diversity per unit area than any other tropical region but has little coverage in studies of island plant biogeography (Raven et al., 2020; Trethowan, 2021).

Here, we examine whether multifactorial environmental stress influences Malesian plant distributions. A recent definition states that stressors are “any deviation in the value of an external environmental … variable from the range of values that is favorable for … an entity” (Love and Wagner, 2022). Here our “entity” is plants, and the “deviation” in question are differences from a seasonal wet tropical lowlands either with altitude, drought, or metal‐rich ultramafic soils. The “favorable” lowland wet tropics enable plant communities to achieve both high biomass and their greatest levels of productivity (Cleveland et al., 2011; Shenkin et al., 2019). Crucially, this simple definition can be applied to large‐scale studies. This differs from the definition used in fine‐scale studies of populations that focuses upon differences from optimum conditions in stressful environments (Love and Wagner, 2022; MacLean et al., 2013), an approach that is less tractable across large scales and many taxa (McGill, 2019). The three stressors focused upon here all have documented examples of how they damage plant function (Zandalinas et al., 2021). First, drought causes hydraulic failure, carbon starvation, and increased pathogen attack and herbivory (Anderegg et al., 2015; Anderegg et al., 2012; Choat et al., 2018; Fensham et al., 2009; McDowell et al., 2008; Powers et al., 2020). Second, ultramafic soils with toxic high metal content damage enzymes, DNA, and cell membranes (Küpper and Andresen, 2016; Singh et al., 2013). Ultramafic soils also have low P, K, and Ca – all key nutrients for plant growth (Proctor, 2003). Likewise, altitude poses difficulties through reductions in temperature and soil fertility (Asner et al., 2014; Grubb, 1977). In Malesia, we can compare the effects of these stressors and dispersal distances between islands upon species distributions (Brambach et al., 2020; Joyce et al., 2020b; Kooyman et al., 2019; Trethowan, 2021).

Traits should influence how plants overcome dispersal distances and environmental stress (Crayn et al., 2015; Grime, 1977; Ottaviani et al., 2020; Schrader et al., 2021; van Steenis, 1962; Yap et al., 2018). There has been evidence from island systems that traits may vary consistently dependent upon the traits of close relatives on the mainland. Known as the “island rule”; traits of insular species with large relatives on the mainland show lower trait values, whereas species with small mainland relatives show increases (Biddick et al., 2019). These changes depend upon the trait in question but also upon the environmental conditions of islands (Biddick et al., 2019; García‐Verdugo et al., 2019). By focusing upon the environmental drivers of species traits, we can hypothesize how traits influence both inter‐island dispersal and toleration of environmental stress. Leaf size and height in tropical ecosystems generally declines when species are better adapted toward stressful environments (Fajardo et al., 2019; Wright et al., 2017). Smaller leaves are less at risk of extreme water loss via transpiration (Wright et al., 2017) and shorter species have smaller conduit size that reduces chance of embolism‐linked death (Olson et al., 2018). Fruit size likely follows a similar pattern whereby low‐productivity high‐stress environments limit the production of large, high‐energy cost fruit (McConkey et al., 2022; Moles et al., 2007). Smaller fruit, however, could also promote dispersal because they can be consumed by both small and large frugivores meaning dispersal is possible via a greater number of agents (Chen and Moles, 2015; Green et al., 2022). Smaller flowers may be advantageous for drought stress tolerance because they lose less water via transpiration (Galen, 1999). Alternatively, because small flowers are more likely to be resource cheap and short‐lived than larger flowers, they could promote dispersal by enabling fast reproduction in newly occupied habitat (Roddy et al., 2021). Small flowers can also be high cost and therefore long‐lived, attracting a greater range of pollinators, increasing the chance of successful pollination in a new area (Roddy et al., 2021). Some traits, therefore, could support both the tolerance of environmental stress and long‐distance dispersal. To address this, we compare how these traits improve the chances of overcoming either stress or the distances between islands. The results will help determine how these traits shape plant distributions at large scales.

The Lamiaceae (mint family) is ideal for studying the drivers of species distributions in Malesia: there are 281 native species, widely distributed across the region and found in almost all vegetation types; plants vary from weedy pioneers to species restricted to mature forest. The family also has substantial variation in stature from herbs to large trees. Crucially, recent floristic work by Bramley et al. (2019) enables accurate species identification, ensuring species distributions and trait data are reliable. Using a dataset built from the description data in Bramley et al. (2019), we examine how traits, dispersal distances, and environmental stress interact to shape Lamiaceae species distribution in Malesia.

## 2 MATERIALS AND METHODS

### 2.1 Study area

Malesia (10° S ‐ 19° N, 94° E ‐ 151° E) is the region spanning the countries of Malaysia, the Philippines, Indonesia, Timor Leste, and Papua New Guinea. Other than mainland Peninsular Malaysia, the region consists of islands separated by seas. Climate varies from the wet tropics to drought‐prone seasonally dry tropics. Altitude shapes habitats from mangroves at sea level to alpine mountain peaks. The region is also home to the tropics' largest area of ultramafic soil/rock which outcrops across most islands (Galey et al., 2017; Garnica‐Díaz et al., 2022).

### 2.2 Malesian Lamiaceae traits

The dataset (Table S1) consists of 222 species in 38 genera. These are all the native species with recorded maximum height, leaf length, leaf width, fruit size, calyx length, and corolla length in Flora Malesiana (Bramley et al., 2019). The 59 native species not analyzed lacked one or more of these recorded measurements. Midpoint leaf length and width (maximum plus minimum values divided by two) are used here alongside maximum values for other traits, because maximum values were often the only measurement recorded in the description. Log‐transformed and scaled (*z*‐scores) species traits were reduced to two principal component (PC) axes via singular value decomposition. The two axes were responsible for 73% variation in traits amongst 222 Lamiaceae species in Malesia (Figure S1). Principal component loadings showed that the first axis corresponds to increasing leaf length (PC axis loading = 0.51), width (PC axis loading = 0.5), and fruit size (PC axis loading = 0.45) and the second a negative correlation between flower size (Calyx length PC axis loading = 0.57, Corolla length PC axis loading = 0.59) and species height (PC axis loading = −0.51) (Figure S2). For simplicity, herein we refer to the two axes as (1) leaf and fruit size and (2) flower size vs height.

### 2.3 Environmental stress

**FIGURE 1:**
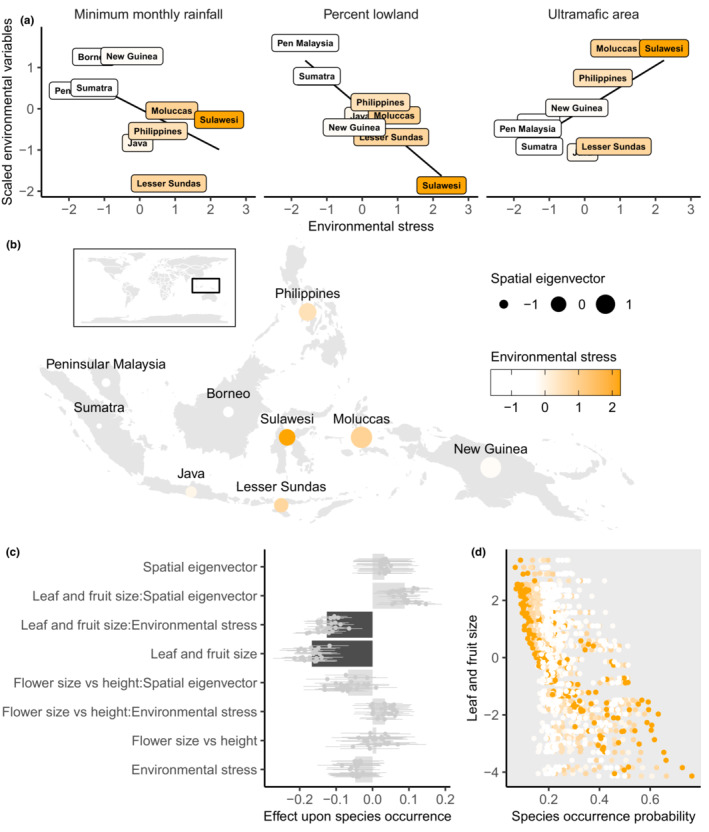
(a) The contributions of ultramafic soils, minimum monthly rainfall, and lowland area to environmental stress – which is a principal component axis that accounts for 53% variation in these variables. (b) Spatial eigenvector scores and environmental stress across taxonomic database working group (tdwg) areas of Malesia and their global position (inset map). (c) Drivers of Lamiaceae species occurrence across Malesia according to phylogenetic generalized mixed effects models. We ran 25 separate models each with a randomly selected number of species equal to the tdwg area of least species richness – each point represents effect score and standard error from each model. Gray and black bars are the mean effect. Bar colors correspond to mean *p* < .05 (black) and mean *p* > .05 (gray). (d) Predicted occurrences of species with varying leaf and fruit size across the environmental stress gradient of Malesian tdwg areas.

Environmental stress was a single PC axis responsible for covariation in increasing ultramafic soil area (PC axis loading = 0.52) and decreasing minimum monthly rainfall (PC axis loading = −0.44) and lowland area (PC axis loading = −0.73) (Figure 1a) in Malesia's nine taxonomic database working group (tdwg) areas: Peninsular Malaysia, Sumatra, Borneo, Philippines, Java, Sulawesi, the Lesser Sundas, Moluccas, and New Guinea (Brummitt, 2001). This axis represented 53% variation in the environmental variables (Figure S3).

Lowland area was the percentage area below 400 m. A 400 m cutoff was chosen because at these altitudes there are noticeable shifts in plant traits and in certain locations in Malesia, montane flora is observed (Holthuis and Lam, 1942; Trethowan, 2021; Umaña and Swenson, 2019). Ultramafic soil area was estimated as the percentage covering tdwg areas from the map presented in Galey et al. (2017). There is currently not an ultramafic soil layer available. Minimum monthly rainfall values were taken from WorldClim, these were values recorded from 1970–2000 (Exposito‐Alonso, 2017) (Table S2). All these variables were scaled prior to the PC analysis. The environmental stress values peak at the archipelago's center, in Sulawesi, Moluccas, Lesser Sundas, and the Philippines, where there is least lowland, most ultramafic soils, and the strongest dry season (Figure 1b).

### 2.4 Environmental stress and Malesian Lamiaceae distributions

To test the effect of environmental stress upon species occurrence, we built the following phylogenetic generalized mixed effects model (Li et al., 2020):

$$Y_{i}∼\text{Bernoulli}\left(p_{i}\right)$$

$$\text{logit}\left(p_{i}\right)=\alpha +\beta \gamma +\delta \gamma +\beta \theta +\delta \theta +a_{\mathrm{spp}\left[i\right]}+b_{\mathrm{spp}\left[i\right]}$$

$$a∼\text{Gaussian}\left(0,\sigma_{a}^{2}I_{n}\right)$$

$$b∼\text{Gaussian}\left(0,\sigma_{b}^{2}\Sigma_{\mathrm{spp}}\right)$$

Greek letters above refer to fixed effects and latin to mixed effects (Gelman and Hill, 2006; Li et al., 2017). Here, $Y_{i}$ represents the observations i of presence/absence n across Malesia's nine tdwg areas m according to Bramley et al. (2019). The logit‐transformed probability of species presence $p_{i}$ was modeled as a function of leaf and fruit size β and flower size vs height δ and their interaction with environmental stress γ and a spatial eigenvector θ. The spatial eigenvector θ was the first selected Moran's eigenvector of spatial distance between tdwg centroids (Dray et al., 2012; Griffith, 1996) (Figure 1b). This involved calculation of a Gabriel neighbor graph between tdwg centroids and subsequent unweighted orthogonalization of the resulting distance matrix (Dray et al., 2012). The intercept α estimates species average presence in tdwg areas.

We included two random effects for species identity: one without $a_{\mathrm{spp}\left[i\right]}$ and one with $b_{\mathrm{spp}\left[i\right]}$ the covariance in species effects decided by phylogenetic distance between them $\sigma_{b}^{2}\Sigma_{\mathrm{spp}}$ (Ives, 2018; Li and Ives, 2017). $\mathrm{spp}\left[i\right]$ connects observations to species. $\Sigma_{\mathrm{spp}}$ represents the n x n phylogenetic distance matrix that assumed a Brownian motion model of evolution and was calculated from a phylogeny built for all species in the dataset (Li et al., 2020). The species random effect without phylogenetic covariance was drawn from a Gaussian distribution with mean 0 and variance $\sigma^{2}$. Phylogenetic data were derived from the latest Lamiaceae backbone (Zhao et al., 2021). Genera missing from the backbone were manually placed, using *phytools* (Revell, 2012), based upon more finescale phylogenetic studies (Li et al., 2016; Steane et al., 2004). Species not in the backbone phylogeny were randomly imputed alongside congeners to produce a bifurcating tree using *pez* (Pearse et al., 2015). This is not expected to affect overall variability in phylogenetic distances between species used in the random effect $b_{\mathrm{spp}\left[i\right]}$ (Li et al., 2019).

To account for more species‐rich areas sharing more species simply because of sample size, we took 25 randomly sampled communities the size of the least species‐rich area and repeated the model for each of these (Cardoso et al., 2009; Nash, 1950; Stier et al., 2016). The averaged effects and Wald test *p* values from these models were used to identify significant effects of predictor variables upon species occurrence.

To further explore model behavior, we extracted predicted occurrences of species. This allowed us to examine species‐predicted occurrence across the phylogeny, tdwg areas, and the environmental stress gradient.

All analyses were carried out in R version 4.0.2.

## 3 RESULTS

Phylogenetic generalized mixed effects models of species presence‐absence across tdwg areas showed that increasing fruit and leaf size had a negative effect upon species occurrence across Malesia (mean effect score = −0.17 and mean Wald test *p* from 25 model iterations < 0.05) (Figure 1c) and that increasing leaf and fruit size decreased the chance that species occurred in areas of high environmental stress (mean effect score = −0.13 and mean Wald test *p* < .05) (Figure 1c). All other predictor variables had considerably lower effect scores none of which were significant (maximum mean effect score = 0.088 all mean *p* > .05, Figure 1c). This indicates that species with smaller fruit and leaves are more likely to occur in areas of high environmental stress in Malesia.

**FIGURE 2:**
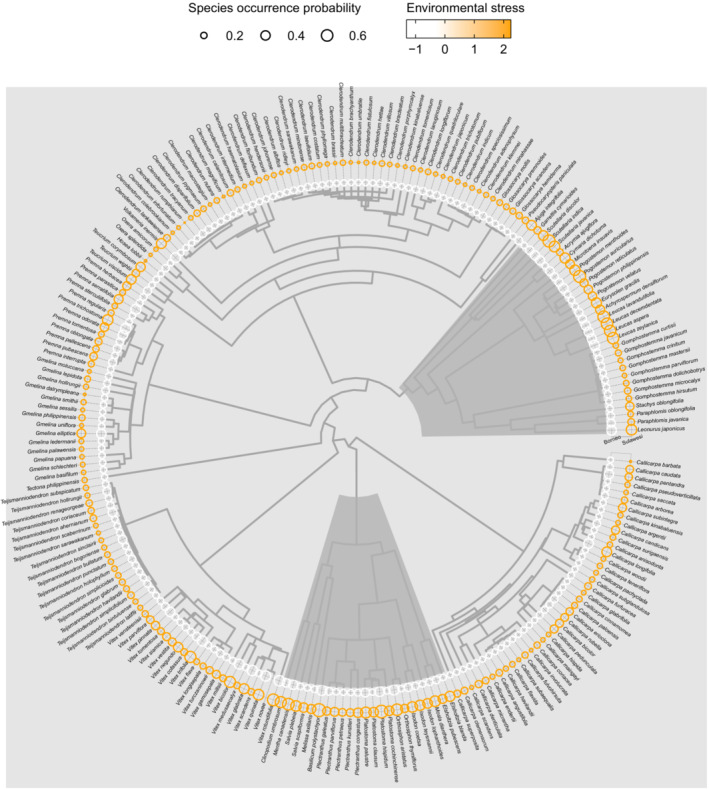
Phylogenetic context of species occurrence probabilities in Borneo and Sulawesi. Two of nine taxonomic working group regions selected to highlight large environmental stress differences between spatially adjacent islands. This also means the probabilities are better visualized than if all data from nine areas were plotted. Species occurrence probabilities are their mean value resulting from the 25 model iterations. Lineages highlighted in dark gray are most diverse in temperate and subtropical regions.

Environmental stress effects upon predicted occurrence vary across the phylogeny. The general pattern being species from clades with greater diversity in the tropics tend to have lower predicted occurrence in high‐stress environments, except for the genera *Vitex* and *Premna* (Figure 2). Species belonging to clades/genera most diverse in temperate and subtropical regions (e.g., *Leucas* and *Salvia*) have consistently high predicted occurrence in stressful environments (Figure 2).

## 4 DISCUSSION

Species occurrence across island communities is often driven by the spatial distance between them (Ibanez et al., 2018; MacArthur and Wilson, 1963). We have shown here that a gradient of environmental stress that represents variation in drought, altitude, and ultramafic soils influence distributions of Lamiaceae species in Malesia (Hulshof and Spasojevic, 2020; Sheldon et al., 2018), whereas previously, plant growth and survival in experimental settings have been shown to be negatively affected by multifactorial stress (Zandalinas et al., 2021), our results show its importance for the distribution of species at large scales. Previous study has shown that low‐temperature stress of temperate regions and high altitude irrespective of latitude drive similarities in plant communities (Segovia et al., 2020). Similar convergence because of additional stressors may occur (Rillig et al., 2019; Zandalinas et al., 2021). For instance, in the Neotropics, stressors include altitude in the Andes, nutrient deficiency of white sands, and drought/fire in the seasonal biome – similarities in how they shape biogeography could be sought (Fine et al., 2014; Pérez‐Escobar et al., 2017; Segovia et al., 2020; Simon et al., 2009).

Our study focuses upon the Lamiaceae; other plant family distributions may or may not follow the pattern we have observed. Firstly, Lamiaceae species have relatively small drupaceous or schizocarpic fruits, families that have larger and different fruit types such as drupes and berries could be more closely linked to frugivore distributions across islands (Crayn et al., 2015; McConkey et al., 2022; McFadden et al., 2022; Yap et al., 2018). Wind‐dispersed seeds may also facilitate patterns different to our observations; noticeably, wind‐dispersed Asteraceae have been shown to be less speciose than expected on islands except when insular diversification has occurred (König et al., 2021). Asteraceae are not particularly species rich in Malesia (Mandel et al., 2019), which suggests that again their dispersal mechanism is not linked to greater insular diversity. Asteraceae versus Lamiaceae also highlights differences in floral morphology. Asteraceae and other families that are more diverse in Malesia, such as the Myrtaceae (Joyce et al., 2020a), have open flowers that differ from the typically tubular, closed flowers of Lamiaceae species. An open flower may enable a wider range of pollinators compared to closed flowers, which could be advantageous in multiple environments with varying pollinator communities (Herrera, 2020).

There has been little clarity as to what factors determine the ability of plant species to colonize new islands and then diversify in Malesia (Shee et al., 2020). We have shown here that environmental stress influences species occurrence: could the ability to tolerate stress also enable access to unoccupied islands and novel conditions – driving diversification (Gavrilets and Losos, 2009; Pillon et al., 2014)? In this study, we demonstrate that a reduction in leaf and fruit size increased the chance that Lamiaceae species were able to cope with environmental stress, allowing them to establish across Malesia's islands. Similarly, non‐Lamiaceae clades may have strategies that allow species to occupy islands across the archipelago. For instance, many clades (e.g., Palms, Cercidoideae, and *Caesalpinia*) that are diverse and widely distributed in Malesia have strategies to cope with stress such as reduced height and stature, often becoming more shrubby or lianescent, compared to species of the same clade outside Malesia (Couvreur et al., 2015; Gagnon et al., 2019; Sinou et al., 2020; Trethowan, 2021; Westoby, 1998). A switch to lianescence could have a positive effect upon dispersal because it increases the opportunity to access tree fall gaps and occupy lower canopied forests where lianas become most abundant (Dalling et al., 2012). By gathering trait data from museum specimens to allow sampling of a high percentage of a clade's species, it should be possible to identify whether traits linked to ecological strategies, such as stress tolerance, encourage dispersal events that precede lineage diversification on islands (Cacho and Strauss, 2014; Esquerré et al., 2020; Heberling and Isaac, 2017). The growing understanding of evolutionary relationships for clades that are speciose in Malesia make examination of this achievable (Atkins et al., 2019; Bellot et al., 2020; Kuhnhäuser et al., 2021; Murphy et al., 2020).

In this study, we have analyzed species rather than lineages, and therefore, we are not able to identify insular speciation events that may influence the patterns we observe. For instance, altitude, drought, and ultramafic soils have been implicated as drivers of insular speciation (Garot et al., 2019; Pillon et al., 2014; Steinbauer et al., 2016). To address this, we need greater sampling of Malesian Lamiaceae species in published phylogenies. However, our results do allow us to survey model predictions of species occurrence across the phylogeny and observe how different clades are affected by tdwg areas with varying environmental stress. What is striking about these results is that the genera *Clerodendrum* and *Teijsmanniodendron*, that have diversified mostly in the tropics, have low predicted occurrence in high‐stress environments, whereas genera that are most diverse in temperate or subtropical regions (*Leucas*, *Salvia*, etc) have consistently high predicted occurrence in stressful environments. This could help inform where we expect to find diversification within islands. For instance, *Vitex*, a speciose tropical genus has high predicted occurrence in stressful environments which could be linked to insular diversification.

Multifactorial environmental stress may help explain diversification more generally. For diversification to occur species must become differentiated. Benign environments without sharp stress gradients lack a potential axis of differentiation (Bouchenak‐Khelladi et al., 2015; Gavrilets and Losos, 2009; Hart and Marshall, 2013). Stress gradients could underpin why mountain ranges with high percentages of ultramafic soils are some of the most diverse regions on earth (Rahbek et al., 2019). There are many examples of diversification across single variable stress gradients. For instance, *Diospyros*, *Codia*, and *Geissosis* diversify across the ultramafic non‐ultramafic soil mosaic of New Caledonia (Paun et al., 2016; Pillon et al., 2014, 2009). Likewise, diversification of the Burseraceae subfamily Protieae is linked to occupation of Neotropical low‐nutrient white sands and flooded forests (Fine et al., 2014). In Malesia, the Lamiaceae genus *Callicarpa* has a center of diversity in the Philippines, islands that have many stressful ultramafic soils, a defined dry season, and many mountains. Philippine montane gradients have been shown to drive mammal diversification (e.g., Heaney et al., 2018). For *Callicarpa*, multifactorial stress, including altitudinal, drought, and soil stress, could be tied to their diversification. Combining variables such as these to identify general environmental stress‐driven diversification would be a tractable approach for studies at local or global scales. This would complement fine‐scale studies showing how stressors can increase population phenotypic plasticity (Levis et al., 2020). Experimental studies also highlight how the effects of stressors are not always consistent between populations or species (Love and Wagner, 2022). Similarly, extratropical clades could experience stress in the warm wet tropics. Therefore, a stress gradient in reverse to that presented in this study could drive diversification of clades with extratropical origins (Baldwin and Wagner, 2010).

This study has not explored human‐caused stressors. Deforestation, non‐natural fires, and domestic livestock‐driven herbivory are all stressors that require examination (Donlan et al., 2002; Gaveau et al., 2021; Nolan et al., 2021; Voigt et al., 2021). Incorporation of these factors alongside climate change predictions will be crucial when modeling future scenarios for the Malesian flora. How species traits affect toleration of anthropogenic stressors, like we have shown for environmental stressors, may prove useful for predicting change on megadiverse islands.

## 5 CONCLUSIONS

Overall, we have identified the importance of environmental stress over large scales. By simplifying Malesia to a gradient of environmental stress, the abiotic influence upon Lamiaceae distributions was clear. Questions remain about how the formation of stressors in relation to rapid island uplift in Malesia has contributed to the diversification of species over time. Comparison of the effects of island and environmental stress formation in diversification rate analyses would address this. Environmental stress could offer an elegant and simple explanation for the distribution and diversity of species.

## AUTHOR CONTRIBUTIONS

**Liam A. Trethowan:** Conceptualization (lead); data curation (equal); formal analysis (lead); writing – original draft (lead); writing – review and editing (equal). **Camilla Arvidsson:** Data curation (equal); writing – review and editing (equal). **Gemma L. C. Bramley:** Conceptualization (equal); data curation (equal); writing – review and editing (equal).

## CONFLICT OF INTEREST

The authors declare no conflict of interest.

## Supporting information


Appendix S1

## Data Availability

Rmarkdown document to build the article and all data, including the phylogenetic tree, is available here: https://figshare.com/projects/Malesian_Mints/149389.
