# Supplementary material for: Environmental stress influences Malesian Lamiaceae distributions
Source: Ecol Evol. 2022 Nov 2;12(11):e9467. doi: 10.1002/ece3.9467 (PMC9627225; doi:10.1002/ece3.9467)
Supplement: Supplementary file 1 — Appendix S1 [file ECE3-12-e9467-s001.docx]

# Supplementary information


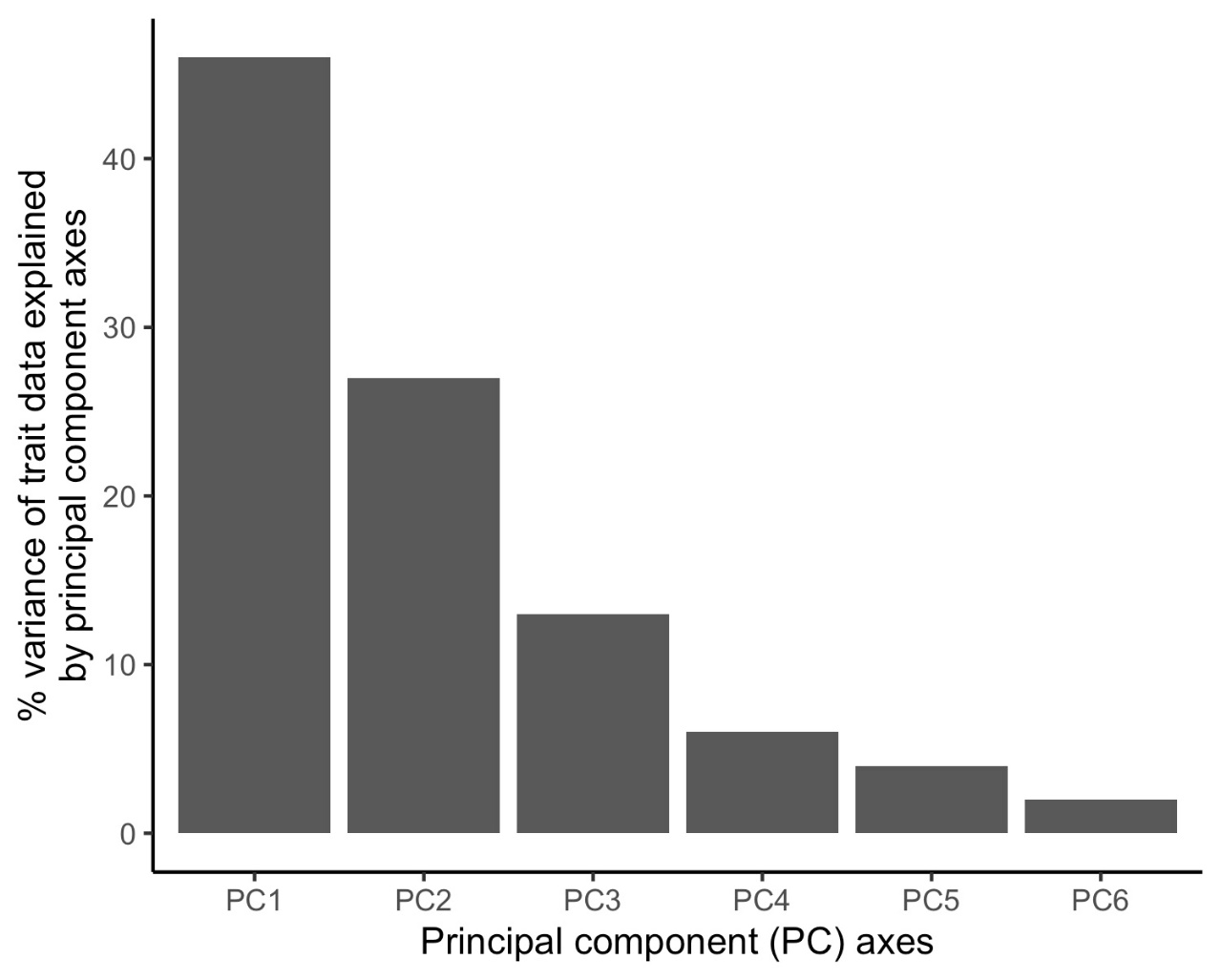


Figure S1: Percentage variation of trait data explained by principal component axes.


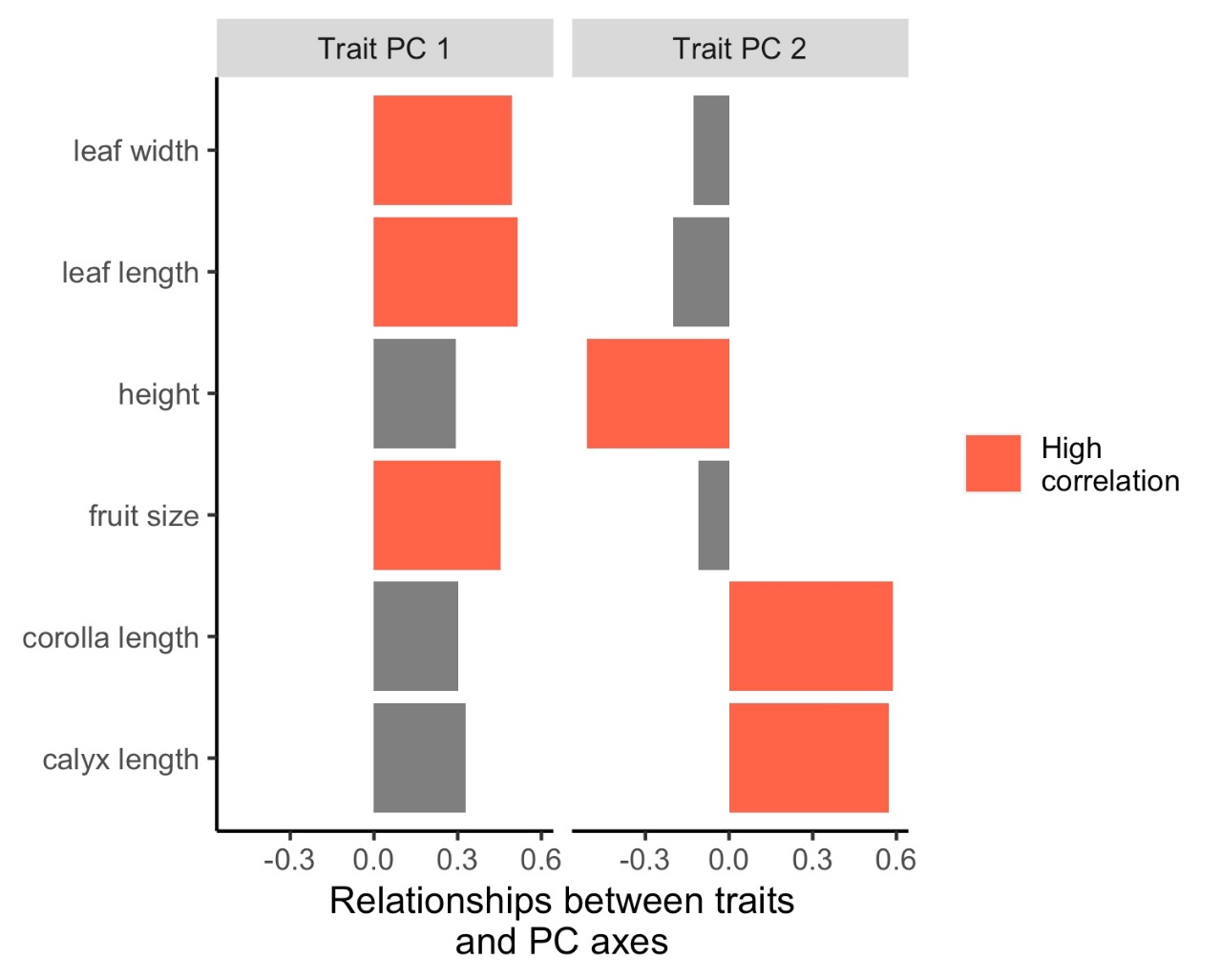


Figure S2: Relationships between Malesian Lamiaceae species traits and trait principal component axes. Correlations > 0.4 and < -0.4 shown by red bars.


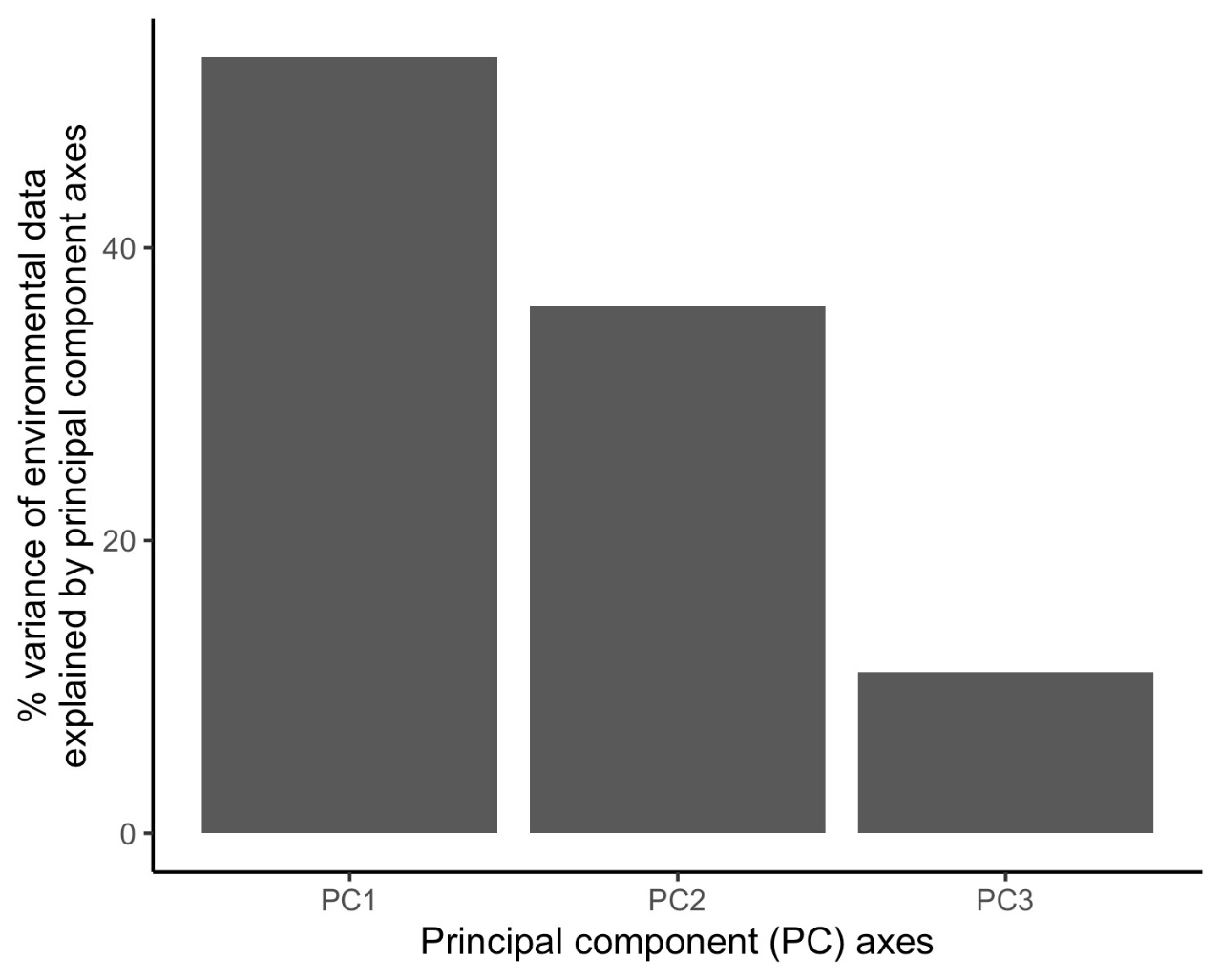


Figure S3: Percentage variation of environmental variables in Malesian taxonomic database working group areas and principal component axes.

Table S1: Malesian Native Lamiaceae species trait data analysed

| Species | Height (mm) | Leaf length (cm) | Leaf width (cm) | Calyx length (mm) | Corolla length (mm) | Fruit size (mm) |
| --- | --- | --- | --- | --- | --- | --- |
| Achyrospermum densiflorum | 60 | 7.00 | 3.25 | 8.0 | 8.0 | 2.00 |
| Acrymia ajugiflora | 10 | 17.50 | 11.00 | 3.0 | 7.0 | 1.50 |
| Ajuga integrifolia | 20 | 6.50 | 2.00 | 5.5 | 8.0 | 2.00 |
| Basilicum polystachyon | 40 | 3.05 | 1.65 | 2.0 | 2.5 | 0.50 |
| Callicarpa angustifolia | 300 | 11.00 | 3.00 | 2.0 | 4.0 | 3.00 |
| Callicarpa anisodonta | 300 | 15.00 | 5.90 | 4.5 | 5.0 | 3.00 |
| Callicarpa arborea | 600 | 18.50 | 9.50 | 1.0 | 4.0 | 3.00 |
| Callicarpa argentii | 300 | 20.00 | 10.25 | 6.0 | 6.0 | 4.00 |
| Callicarpa barbata | 800 | 42.50 | 23.00 | 3.0 | 7.0 | 7.00 |
| Callicarpa bicolor | 400 | 9.00 | 4.35 | 1.0 | 3.0 | 2.00 |
| Callicarpa candicans | 400 | 11.00 | 6.25 | 1.0 | 3.0 | 2.00 |
| Callicarpa caudata | 600 | 12.00 | 3.75 | 1.0 | 3.0 | 2.00 |
| Callicarpa cinnamomea | 500 | 12.00 | 5.10 | 3.0 | 6.0 | 3.50 |
| Callicarpa clemensorum | 3 | 14.00 | 7.00 | 4.0 | 5.0 | 4.00 |
| Callicarpa coriacea | 3 | 15.25 | 8.75 | 6.0 | 7.0 | 4.00 |
| Callicarpa denticulata | 3 | 15.00 | 9.75 | 1.5 | 3.0 | 2.00 |
| Callicarpa endertii | 300 | 16.50 | 8.50 | 4.0 | 4.0 | 4.00 |
| Callicarpa erioclona | 900 | 18.25 | 7.00 | 1.0 | 2.5 | 2.00 |
| Callicarpa flavida | 1000 | 13.50 | 4.00 | 3.0 | 5.0 | 4.00 |
| Callicarpa fulvohirsuta | 500 | 11.00 | 4.50 | 1.5 | 3.0 | 4.00 |
| Callicarpa furfuracea | 300 | 15.50 | 6.50 | 1.0 | 4.0 | 3.00 |
| Callicarpa glabrifolia | 800 | 22.00 | 7.75 | 2.0 | 5.0 | 4.00 |
| Callicarpa havilandii | 800 | 18.50 | 5.50 | 2.0 | 5.0 | 4.00 |
| Callicarpa hispida | 600 | 15.00 | 7.00 | 5.0 | 7.0 | 7.00 |
| Callicarpa involucrata | 1000 | 29.50 | 10.25 | 5.0 | 6.0 | 5.00 |
| Callicarpa kinabaluensis | 150 | 15.00 | 7.75 | 5.0 | 8.0 | 3.00 |
| Callicarpa longifolia | 800 | 13.50 | 6.00 | 1.0 | 2.5 | 3.00 |
| Callicarpa maingayi | 1500 | 22.50 | 12.50 | 1.5 | 4.0 | 3.00 |
| Callicarpa micrantha | 600 | 11.25 | 3.50 | 1.0 | 3.0 | 3.00 |
| Callicarpa pachyclada | 300 | 28.25 | 19.50 | 1.5 | 2.5 | 2.00 |
| Callicarpa paloensis | 900 | 13.25 | 5.00 | 1.0 | 2.5 | 4.00 |
| Callicarpa pedunculata | 500 | 8.00 | 3.95 | 1.0 | 3.0 | 2.00 |
| Callicarpa pentandra | 2000 | 20.00 | 9.50 | 3.0 | 6.0 | 5.00 |
| Callicarpa pseudoverticillata | 600 | 14.25 | 6.50 | 2.0 | 7.0 | 4.00 |
| Callicarpa rubella | 300 | 13.50 | 3.50 | 1.2 | 2.7 | 3.50 |
| Callicarpa saccata | 500 | 18.50 | 8.25 | 5.0 | 7.0 | 5.00 |
| Callicarpa scandens | 600 | 36.50 | 16.00 | 1.0 | 5.0 | 3.00 |
| Callicarpa subaequalis | 500 | 20.00 | 11.50 | 5.0 | 5.0 | 5.00 |
| Callicarpa subglandulosa | 1500 | 11.50 | 6.00 | 2.5 | 5.0 | 4.00 |
| Callicarpa subintegra | 500 | 7.10 | 1.50 | 1.5 | 2.5 | 2.00 |
| Callicarpa superposita | 500 | 12.75 | 3.50 | 1.0 | 2.5 | 2.00 |
| Callicarpa surigaensis | 800 | 13.25 | 4.35 | 2.0 | 5.0 | 4.00 |
| Callicarpa teneriflora | 500 | 19.00 | 7.50 | 1.0 | 3.5 | 3.00 |
| Callicarpa woodii | 600 | 19.25 | 6.50 | 2.0 | 6.0 | 6.00 |
| Clerodendrum adenophysum | 200 | 22.00 | 18.50 | 7.0 | 17.0 | 9.00 |
| Clerodendrum albiflos | 500 | 23.50 | 8.50 | 4.0 | 15.0 | 9.00 |
| Clerodendrum brachyanthum | 400 | 24.00 | 15.50 | 6.0 | 9.0 | 7.00 |
| Clerodendrum bracteatum | 800 | 13.50 | 9.00 | 7.0 | 3.8 | 8.00 |
| Clerodendrum brassii | 300 | 20.00 | 11.50 | 19.0 | 21.0 | 8.00 |
| Clerodendrum calamitosum | 50 | 10.00 | 6.50 | 7.0 | 25.0 | 6.00 |
| Clerodendrum colebrookianum | 600 | 17.50 | 16.25 | 7.0 | 25.0 | 7.00 |
| Clerodendrum costatum | 400 | 12.75 | 8.50 | 9.0 | 8.0 | 6.00 |
| Clerodendrum deflexum | 300 | 26.00 | 9.75 | 9.0 | 23.0 | 8.00 |
| Clerodendrum disparifolium | 200 | 12.00 | 5.00 | 7.0 | 24.0 | 6.00 |
| Clerodendrum fistulosum | 100 | 20.00 | 6.25 | 8.0 | 9.0 | 7.00 |
| Clerodendrum floribundum | 800 | 9.00 | 6.00 | 6.0 | 35.0 | 4.50 |
| Clerodendrum haematolasium | 400 | 22.00 | 11.00 | 9.5 | 13.0 | 6.00 |
| Clerodendrum hendersonii | 400 | 14.50 | 7.75 | 7.0 | 22.0 | 8.00 |
| Clerodendrum hettae | 200 | 18.50 | 13.50 | 22.0 | 31.0 | 7.00 |
| Clerodendrum indicum | 300 | 15.50 | 1.90 | 8.0 | 90.0 | 120.00 |
| Clerodendrum infortunatum | 800 | 16.50 | 13.00 | 9.0 | 7.0 | 8.00 |
| Clerodendrum intermedium | 200 | 18.00 | 14.50 | 6.0 | 9.0 | 8.00 |
| Clerodendrum japonicum | 900 | 25.50 | 24.00 | 9.0 | 20.0 | 8.00 |
| Clerodendrum johorense | 300 | 23.75 | 3.65 | 7.0 | 18.0 | 4.00 |
| Clerodendrum kinabaluense | 150 | 20.00 | 9.00 | 18.0 | 18.0 | 8.50 |
| Clerodendrum klemmei | 500 | 12.50 | 5.00 | 8.0 | 6.0 | 9.00 |
| Clerodendrum laevifolium | 250 | 13.50 | 4.10 | 8.0 | 13.0 | 6.00 |
| Clerodendrum lankawiense | 25 | 13.25 | 2.75 | 8.0 | 3.0 | 6.00 |
| Clerodendrum lanuginosum | 700 | 16.00 | 13.25 | 17.0 | 21.0 | 9.00 |
| Clerodendrum longiflorum | 900 | 15.00 | 7.75 | 8.0 | 8.2 | 6.00 |
| Clerodendrum macrostegium | 600 | 20.00 | 12.50 | 18.0 | 31.0 | 7.00 |
| Clerodendrum magnificum | 300 | 20.50 | 12.00 | 25.0 | 26.0 | 8.00 |
| Clerodendrum minahassae | 400 | 21.00 | 10.25 | 37.0 | 50.0 | 9.00 |
| Clerodendrum mindorense | 400 | 14.50 | 5.50 | 5.0 | 30.0 | 9.00 |
| Clerodendrum multibracteatum | 400 | 11.75 | 4.50 | 7.0 | 7.0 | 6.00 |
| Clerodendrum myrmecophilum | 150 | 22.50 | 11.00 | 8.0 | 23.0 | 7.50 |
| Clerodendrum nutans | 300 | 14.50 | 5.00 | 7.0 | 8.0 | 8.50 |
| Clerodendrum phyllomega | 200 | 29.50 | 10.00 | 9.0 | 23.0 | 7.50 |
| Clerodendrum porphyrocalyx | 600 | 21.00 | 7.50 | 7.0 | 20.0 | 9.50 |
| Clerodendrum pubiflorum | 600 | 8.50 | 9.00 | 6.5 | 16.0 | 12.00 |
| Clerodendrum pygmaeum | 5 | 12.50 | 8.00 | 18.0 | 14.0 | 8.00 |
| Clerodendrum quadriloculare | 400 | 16.50 | 6.50 | 18.0 | 45.0 | 9.00 |
| Clerodendrum ridleyi | 500 | 19.50 | 4.25 | 21.0 | 80.0 | 9.50 |
| Clerodendrum rumphianum | 200 | 26.00 | 22.50 | 20.0 | 40.0 | 8.00 |
| Clerodendrum sarawakanum | 90 | 16.00 | 9.50 | 16.0 | 15.0 | 8.00 |
| Clerodendrum speciosissimum | 400 | 22.00 | 17.00 | 4.0 | 27.0 | 8.00 |
| Clerodendrum tomentosum | 700 | 10.00 | 4.25 | 9.0 | 25.0 | 9.00 |
| Clerodendrum tracyanum | 2000 | 21.50 | 15.50 | 9.0 | 8.0 | 9.00 |
| Clerodendrum trichotomum | 300 | 11.25 | 7.75 | 13.0 | 20.0 | 7.00 |
| Clerodendrum umbratile | 500 | 30.00 | 10.00 | 14.0 | 14.0 | 12.00 |
| Clerodendrum villosum | 500 | 14.50 | 9.50 | 7.0 | 7.0 | 8.00 |
| Clinopodium umbrosum | 25 | 2.75 | 1.90 | 9.0 | 5.0 | 1.00 |
| Cymaria dichotoma | 50 | 8.00 | 4.75 | 1.5 | 2.5 | 1.50 |
| Elsholtzia blanda | 100 | 4.00 | 0.75 | 2.0 | 3.0 | 0.70 |
| Elsholtzia pubescens | 200 | 6.50 | 3.00 | 4.5 | 7.0 | 1.20 |
| Eurysolen gracilis | 30 | 7.00 | 3.25 | 3.5 | 6.5 | 1.00 |
| Garrettia cymarioides | 200 | 6.00 | 2.25 | 1.7 | 1.7 | 20.00 |
| Glossocarya hemiderma | 700 | 6.75 | 4.00 | 3.0 | 4.0 | 7.50 |
| Glossocarya mollis | 600 | 8.75 | 7.50 | 3.5 | 6.5 | 6.00 |
| Glossocarya premnoides | 700 | 7.00 | 5.50 | 3.5 | 8.0 | 7.50 |
| Glossocarya scandens | 700 | 8.50 | 5.25 | 3.0 | 5.0 | 5.00 |
| Gmelina basifilum | 700 | 15.00 | 6.75 | 4.5 | 5.0 | 30.00 |
| Gmelina dalrympleana | 4000 | 21.50 | 14.00 | 6.0 | 22.0 | 8.00 |
| Gmelina elliptica | 800 | 8.00 | 5.00 | 5.0 | 16.0 | 8.00 |
| Gmelina hollrungii | 600 | 23.00 | 15.00 | 5.0 | 7.0 | 8.00 |
| Gmelina ledermanii | 2000 | 13.50 | 7.50 | 5.0 | 7.5 | 6.00 |
| Gmelina lepidota | 750 | 11.00 | 4.65 | 3.5 | 9.0 | 9.00 |
| Gmelina moluccana | 900 | 25.50 | 13.00 | 5.0 | 8.0 | 18.00 |
| Gmelina palawensis | 500 | 14.00 | 6.25 | 5.0 | 13.0 | 22.00 |
| Gmelina papuana | 700 | 19.00 | 9.25 | 5.0 | 6.0 | 7.00 |
| Gmelina philippinensis | 800 | 6.75 | 3.20 | 4.0 | 16.0 | 9.00 |
| Gmelina schlechteri | 600 | 16.50 | 9.50 | 3.0 | 9.0 | 9.00 |
| Gmelina sessilis | 600 | 19.00 | 14.50 | 6.0 | 6.0 | 12.00 |
| Gmelina smithii | 4500 | 10.25 | 6.00 | 5.0 | 6.0 | 7.00 |
| Gmelina uniflora | 400 | 15.00 | 13.25 | 20.0 | 32.0 | 4.00 |
| Gomphostemma crinitum | 50 | 24.00 | 8.00 | 15.0 | 35.0 | 6.00 |
| Gomphostemma curtisii | 90 | 10.00 | 9.00 | 20.0 | 38.0 | 6.00 |
| Gomphostemma dolichobotrys | 200 | 16.50 | 10.00 | 7.0 | 20.0 | 1.00 |
| Gomphostemma hirsutum | 70 | 20.50 | 4.50 | 17.0 | 45.0 | 1.80 |
| Gomphostemma javanicum | 50 | 21.50 | 8.75 | 7.0 | 55.0 | 6.00 |
| Gomphostemma mastersii | 35 | 9.50 | 6.00 | 22.0 | 55.0 | 6.00 |
| Gomphostemma microcalyx | 60 | 16.00 | 7.25 | 7.0 | 20.0 | 2.50 |
| Gomphostemma parviflorum | 300 | 17.50 | 8.00 | 14.0 | 25.0 | 8.00 |
| Hosea lobbii | 800 | 11.00 | 7.25 | 12.0 | 20.0 | 80.00 |
| Isodon coetsa | 80 | 5.00 | 3.00 | 2.0 | 5.0 | 1.00 |
| Isodon lophanthoides | 100 | 2.75 | 1.90 | 2.0 | 6.0 | 1.00 |
| Isodon teysmannii | 50 | 3.75 | 2.50 | 2.0 | 6.0 | 1.00 |
| Leonurus japonicus | 50 | 6.00 | 3.75 | 8.0 | 14.0 | 3.00 |
| Leucas aspera | 60 | 4.75 | 0.80 | 9.0 | 11.0 | 2.50 |
| Leucas decemdentata | 25 | 4.25 | 2.75 | 4.0 | 8.0 | 1.50 |
| Leucas lavandulifolia | 80 | 4.25 | 0.70 | 7.0 | 10.0 | 2.50 |
| Leucas zeylanica | 60 | 5.00 | 1.75 | 6.0 | 9.0 | 3.00 |
| Melissa axillaris | 100 | 2.10 | 1.15 | 6.0 | 9.0 | 8.00 |
| Mentha canadensis | 60 | 4.75 | 2.00 | 2.5 | 5.0 | 1.00 |
| Microtoena insuavis | 50 | 8.50 | 6.00 | 2.5 | 9.0 | 1.50 |
| Mosla dianthera | 100 | 2.25 | 1.50 | 2.0 | 3.5 | 1.00 |
| Orthosiphon aristatus | 25 | 6.00 | 2.60 | 5.0 | 20.0 | 2.00 |
| Orthosiphon thymiflorus | 50 | 5.00 | 3.50 | 5.0 | 14.0 | 1.20 |
| Oxera amicorum | 300 | 12.60 | 7.60 | 6.0 | 60.0 | 9.00 |
| Oxera splendida | 600 | 19.00 | 10.75 | 8.0 | 76.0 | 60.00 |
| Paraphlomis javanica | 50 | 19.50 | 8.10 | 8.0 | 25.0 | 6.00 |
| Paraphlomis oblongifolia | 200 | 17.50 | 7.00 | 12.0 | 20.0 | 8.00 |
| Platostoma clausum | 30 | 5.25 | 1.50 | 3.0 | 4.0 | 1.00 |
| Platostoma cochinchinense | 50 | 5.00 | 1.25 | 3.0 | 4.0 | 1.00 |
| Platostoma hispidum | 100 | 3.75 | 0.75 | 2.5 | 3.0 | 1.00 |
| Platostoma palustre | 50 | 5.00 | 2.35 | 3.0 | 4.0 | 1.50 |
| Plectranthus congestus | 150 | 4.00 | 2.75 | 2.0 | 6.0 | 0.80 |
| Plectranthus galeatus | 150 | 12.00 | 8.00 | 5.0 | 2.0 | 1.00 |
| Plectranthus kunstleri | 50 | 9.00 | 5.00 | 2.5 | 7.0 | 1.00 |
| Plectranthus parviflorus | 100 | 5.00 | 2.75 | 2.5 | 5.5 | 0.80 |
| Plectranthus petraeus | 150 | 7.00 | 4.50 | 3.5 | 6.0 | 1.00 |
| Pogostemon auricularius | 80 | 5.00 | 2.50 | 1.5 | 2.5 | 0.60 |
| Pogostemon menthoides | 50 | 4.50 | 2.85 | 4.5 | 7.0 | 0.60 |
| Pogostemon philippinensis | 75 | 6.00 | 3.75 | 6.0 | 7.0 | 0.70 |
| Pogostemon reticulatus | 75 | 7.75 | 5.75 | 6.0 | 7.0 | 0.75 |
| Pogostemon velatus | 100 | 7.00 | 3.25 | 5.0 | 8.0 | 0.60 |
| Premna herbacea | 5 | 7.75 | 6.00 | 2.5 | 2.0 | 8.00 |
| Premna interrupta | 400 | 17.00 | 8.00 | 3.0 | 2.0 | 2.50 |
| Premna oblongata | 1800 | 16.50 | 10.50 | 2.0 | 2.5 | 4.00 |
| Premna odorata | 2500 | 11.25 | 7.75 | 3.0 | 3.0 | 5.00 |
| Premna pallescens | 600 | 14.50 | 5.00 | 2.2 | 1.8 | 4.50 |
| Premna parasitica | 500 | 19.50 | 13.00 | 2.2 | 3.5 | 4.00 |
| Premna pubescens | 800 | 10.00 | 6.00 | 3.0 | 5.0 | 5.00 |
| Premna regularis | 500 | 13.25 | 9.50 | 1.7 | 3.2 | 5.00 |
| Premna serratifolia | 1000 | 11.50 | 8.50 | 2.0 | 5.0 | 8.00 |
| Premna sterculiifolia | 700 | 13.00 | 11.00 | 2.0 | 4.0 | 4.50 |
| Premna tomentosa | 500 | 19.25 | 14.50 | 3.0 | 4.0 | 6.50 |
| Premna trichostoma | 200 | 10.75 | 5.75 | 1.8 | 3.0 | 5.00 |
| Pseudocaryopteris paniculata | 300 | 11.50 | 4.25 | 2.0 | 3.0 | 4.00 |
| Salvia plebeia | 60 | 3.00 | 1.15 | 2.0 | 3.0 | 1.00 |
| Salvia scapiformis | 20 | 2.50 | 1.60 | 5.5 | 9.0 | 2.50 |
| Scutellaria discolor | 20 | 6.75 | 6.25 | 2.5 | 12.0 | 1.20 |
| Scutellaria indica | 30 | 2.25 | 1.75 | 1.5 | 14.0 | 1.00 |
| Scutellaria javanica | 60 | 5.25 | 2.50 | 4.0 | 16.0 | 1.50 |
| Stachys oblongifolia | 60 | 5.00 | 2.00 | 7.0 | 12.0 | 1.80 |
| Tectona philippinensis | 1500 | 10.50 | 4.50 | 5.0 | 8.0 | 8.00 |
| Teijsmanniodendron ahernianum | 900 | 21.00 | 7.50 | 3.0 | 8.0 | 20.00 |
| Teijsmanniodendron bintuluense | 850 | 21.00 | 6.50 | 5.5 | 3.5 | 5.00 |
| Teijsmanniodendron bogoriense | 4500 | 17.00 | 6.50 | 25.0 | 2.0 | 80.00 |
| Teijsmanniodendron bullatum | 1200 | 16.25 | 5.75 | 3.0 | 2.0 | 5.00 |
| Teijsmanniodendron coriaceum | 400 | 12.50 | 4.50 | 15.0 | 6.0 | 8.00 |
| Teijsmanniodendron glabrum | 400 | 12.00 | 5.50 | 3.0 | 5.0 | 8.00 |
| Teijsmanniodendron havilandii | 800 | 10.00 | 4.50 | 3.0 | 5.0 | 9.00 |
| Teijsmanniodendron holophyllum | 3000 | 23.50 | 8.00 | 2.5 | 4.0 | 9.00 |
| Teijsmanniodendron hollrungii | 2500 | 25.00 | 9.00 | 4.0 | 7.0 | 20.00 |
| Teijsmanniodendron latiffii | 500 | 30.00 | 7.50 | 4.0 | 2.5 | 5.00 |
| Teijsmanniodendron pteropodum | 600 | 32.50 | 11.00 | 2.5 | 7.0 | 50.00 |
| Teijsmanniodendron punctatum | 800 | 14.00 | 5.00 | 2.5 | 3.5 | 15.00 |
| Teijsmanniodendron renageorgeae | 400 | 9.00 | 2.50 | 2.5 | 3.0 | 35.00 |
| Teijsmanniodendron sarawakanum | 4000 | 19.25 | 6.50 | 2.5 | 3.5 | 25.00 |
| Teijsmanniodendron scaberrinum | 2000 | 16.50 | 8.00 | 3.0 | 7.0 | 9.00 |
| Teijsmanniodendron simplicifolium | 500 | 10.00 | 3.50 | 2.0 | 2.5 | 20.00 |
| Teijsmanniodendron simplicioides | 6000 | 14.00 | 5.50 | 2.0 | 5.0 | 6.00 |
| Teijsmanniodendron sinclairii | 300 | 21.00 | 10.50 | 3.0 | 3.8 | 17.00 |
| Teijsmanniodendron subspicatum | 300 | 23.00 | 9.50 | 2.0 | 3.5 | 9.00 |
| Teijsmanniodendron unifoliolatum | 2600 | 26.00 | 7.50 | 3.0 | 5.0 | 7.00 |
| Teijsmanniodendron zainudinii | 2500 | 8.00 | 4.00 | 2.0 | 5.5 | 17.00 |
| Teucrium corymbosum | 100 | 3.75 | 1.40 | 2.5 | 6.0 | 1.20 |
| Teucrium viscidum | 80 | 7.00 | 3.50 | 3.0 | 5.0 | 1.50 |
| Teucrium wightii | 50 | 7.50 | 3.00 | 5.0 | 20.0 | 1.50 |
| Vitex bicolor | 500 | 5.40 | 1.95 | 1.5 | 6.2 | 5.80 |
| Vitex cofassus | 6000 | 16.25 | 5.90 | 2.3 | 5.0 | 7.50 |
| Vitex flava | 500 | 11.50 | 5.50 | 4.0 | 5.0 | 7.00 |
| Vitex gamosepala | 400 | 9.25 | 2.75 | 0.5 | 4.0 | 6.00 |
| Vitex glabrata | 2500 | 10.50 | 4.25 | 2.5 | 6.5 | 3.00 |
| Vitex longisepala | 300 | 12.25 | 4.40 | 5.0 | 7.0 | 6.00 |
| Vitex medusaecalyx | 650 | 9.25 | 4.00 | 5.0 | 5.0 | 6.00 |
| Vitex millsii | 700 | 8.50 | 4.20 | 2.0 | 8.0 | 6.00 |
| Vitex negundo | 400 | 3.65 | 1.10 | 1.5 | 3.5 | 4.00 |
| Vitex novae-pommeraniae | 750 | 12.25 | 5.50 | 3.0 | 6.0 | 9.00 |
| Vitex parviflora | 400 | 10.75 | 3.75 | 2.0 | 6.0 | 8.00 |
| Vitex pinnata | 2500 | 10.50 | 5.95 | 4.0 | 6.0 | 7.50 |
| Vitex quinata | 400 | 6.40 | 2.70 | 3.0 | 6.0 | 8.00 |
| Vitex rotundifolia | 60 | 3.35 | 2.05 | 4.0 | 8.3 | 5.30 |
| Vitex scandens | 300 | 11.25 | 5.50 | 5.0 | 5.0 | 3.00 |
| Vitex siamica | 1800 | 4.95 | 1.90 | 2.0 | 2.5 | 5.00 |
| Vitex tomentosa | 150 | 11.00 | 3.75 | 2.7 | 4.0 | 8.50 |
| Vitex trifolia | 800 | 4.50 | 1.55 | 3.0 | 7.0 | 6.20 |
| Vitex turczaninowii | 900 | 9.50 | 4.20 | 3.0 | 5.0 | 7.00 |
| Vitex vansteenisii | 850 | 9.75 | 4.50 | 3.0 | 5.0 | 6.00 |
| Vitex vestita | 250 | 7.00 | 2.25 | 5.0 | 6.5 | 6.00 |
| Volkameria inermis | 300 | 6.50 | 3.00 | 4.0 | 30.0 | 8.00 |

Table S2: Environmental data for the taxonomic database working group (tdwg) areas of Malesia

| tdwg area | Minimum monthly rainfall (mm) | Percentage area below 400 m | Percentage ultramafic area |
| --- | --- | --- | --- |
| Borneo | 163 | 77 | 10 |
| Java | 62 | 69 | 0 |
| Lesser Sundas | 15 | 64 | 2 |
| Moluccas | 100 | 69 | 35 |
| New Guinea | 164 | 66 | 15 |
| Peninsular Malaysia | 123 | 84 | 8 |
| Philippines | 76 | 71 | 25 |
| Sulawesi | 89 | 54 | 35 |
| Sumatra | 127 | 77 | 2 |
